# Investigating the Targets of *MIR-15a* and *MIR-16-1* in Patients with Chronic Lymphocytic Leukemia (CLL)

**DOI:** 10.1371/journal.pone.0007169

**Published:** 2009-09-25

**Authors:** Katy Hanlon, Claudius E. Rudin, Lorna W. Harries

**Affiliations:** 1 Department of Haematology, Royal Devon and Exeter NHS Foundation Trust, Exeter, Devon, United Kingdom; 2 Molecular Genetics, Royal Devon and Exeter NHS Foundation Trust, Exeter, Devon, United Kingdom; 3 Institute of Biomedical and Clinical Sciences, Peninsula Medical School, Exeter, Devon, United Kingdom; Texas Tech University Health Sciences Center, United States of America

## Abstract

**Background:**

MicroRNAs (miRNAs) are short, noncoding RNAs that regulate the expression of multiple target genes. Deregulation of miRNAs is common in human tumorigenesis. The miRNAs, *MIR-15a/16-1*, at chromosome band 13q14 are down-regulated in the majority of patients with chronic lymphocytic leukaemia (CLL).

**Methodology/Principal Findings:**

We have measured the expression of *MIR-15a/16-1*, and 92 computationally-predicted *MIR-15a/16-1* target genes in CLL patients and in normal controls. We identified 35 genes that are deregulated in CLL patients, 5 of which appear to be specific targets of the *MIR-15a/16-1* cluster. These targets included *2* genes (*BAZ2A* and *RNF41*) that were significantly up-regulated (p<0.05) and 3 genes (*RASSF5, MKK3* and *LRIG1*) that were significantly down-regulated (p<0.05) in CLL patients with down-regulated *MIR-15a/16-1* expression.

**Significance:**

The genes identified here as being subject to *MIR-15a/16-1* regulation could represent direct or indirect targets of these miRNAs. Many of these are good biological candidates for involvement in tumorigenesis and as such, may be important in the aetiology of CLL.

## Introduction

Chronic lymphocytic leukemia (CLL) is the most common adult leukemia in the Western world. It is a heterogeneous disease associated with a highly variable clinical course [1]. A key feature of CLL is cytogenetic instability, with chromosomal abnormalities occurring in around 80% of cases [2]. While the molecular aetiology of CLL remains largely undetermined, specific recurrent chromosomal aberrations have been well described and serve as independent prognostic indicators for disease progression and survival [2].

Deletion of chromosome 13q is the most frequent chromosomal aberration in CLL, occurring in approximately 50% of patients [2]. The deletion of chromosome band 13q14 has also been reported in a variety of other malignancies [3]–[5], demonstrating the importance of this region in tumorigenesis. There has been wide speculation that the 13q14 region harbours tumour suppressor gene(s) involved in the aetiology of these diseases [6], [7]. Various candidate tumour suppressor genes within the minimal deleted region (MDR) at 13q14 have been investigated, yet studies have consistently failed to detect any pathogenic mutations [8]–[10]. There remains, therefore, a need to identify alternative mechanisms that may influence the development of CLL.

There is increasing evidence for the involvement of microRNAs (miRNAs) in tumorigenesis [11]–[15]. MicroRNAs are small, non-coding RNAs that mediate the expression of target genes through sequence-specific base pairing with target messenger RNA (mRNA) [16]. Target gene expression is regulated by the degradation of the mRNA or more commonly, through blocking translation [17]–[19]. Deregulation of miRNAs has been implicated in human tumorigenesis and many miRNAs are located in genomic regions involved in cancer [20].

Two miRNAs, *MIR-15a* and *MIR-16-1*, are located at chromosome band 13q14 and are down-regulated in the majority of patients with CLL [21]. These genes induce apoptosis through the negative regulation of the anti-apoptotic gene *BCL2* [22]. As such, down-regulation of *MIR-15a/16-1* has been associated with the pathogenesis of CLL, although this remains controversial [23].

Each miRNA has the potential to mediate the expression of many target genes [19]. It is therefore possible that *MIR-15a/16-1* may regulate the expression of genes, other than *BCL2*, which may be important in the development of CLL. Indeed, a recent study combining experimental and bioinformatic data identified a *MIR-15a/16-1* gene signature in leukaemic cells [24]. The aim of this study was to examine the expression patterns of computationally-predicted targets of *MIR-15a/16-1* to identify further novel candidate genes involved in the aetiology of CLL.

## Materials and Methods

### Ethics Statement

Ethical approval was granted by the North and East Devon Local Research Ethics Committee and all patients provided written informed consent in accordance with the Declaration of Helsinki.

### Patients

Thirteen patients with CLL under the care of the Haematology Unit at the Royal Devon and Exeter Hospital were enrolled in this study. Each patient had an established diagnosis of CLL based on current World Health Organization (WHO) classification guidelines [25]. Patients had a history of persistent lymphocytosis >5×10^9^/l and an immunophenotypic profile typical of CLL [26]. The majority of patients (12/13) had received treatment for their disease prior to involvement in this study. Of the 13 CLL patients included in this study, 7 (54%) had a deletion at 13q14 confirmed by fluorescence *in situ* hybridisation (FISH) and multiplex ligation-dependent probe amplification (MLPA) (data not shown). Five normal control samples from healthy volunteers were also obtained for this study.

### RNA Extraction

Total RNA was extracted from whole blood using the MirVana miRNA Isolation kit (Ambion/Applied Biosystems, Foster City, USA), according to the manufacturer's instructions. RNA concentrations were measured using a ND-1000 spectrophotometer (NanoDrop Technologies, Wilmington, USA).

### MicroRNA-15a/16-1Expression

*MicroRNA-15a/16-1* expression was determined for each sample using the TaqMan MicroRNA assays kit (Applied Biosystems, Foster City, USA), according to the manufacturer's instructions. The protocol involves real-time quantification of microRNAs by stem-loop RT-PCR, as previously described [27]. All PCR reactions were performed in triplicate. Crossing points (Ct) were determined for each *miRNA* and an endogenous control gene (beta glucorinidase (*GUSB*). The relative abundance of each *miRNA* transcript was then determined using the comparative Ct method [28]. Down-regulated *miR-15a/16-1* expression in CLL patients was defined as a ≥50% reduction in the expression levels of either *MIR-15a* or *MIR-16-1* when compared with the average *MIR-15a* and *MIR-16-1* expression levels in the normal control subjects.

### Tiled Low Density Array (TLDA) Analysis

#### Reverse Transcription

Total RNA (1 µg) was reversed transcribed in 50 µl reactions using the TaqMan Reverse Transcription kit (Applied Biosystems, Foster City, USA), according to the manufacturer's instructions. PCR amplification of the endogenous *GUSB* gene was performed to ensure that the resulting cDNA samples were of adequate quality. PCR reactions were performed in triplicate on the ABI 7900HT Prism (Applied Biosystems, Foster City, USA). Probe and primer sequences are available on request. Each 25 µl reaction included 5 µl universal master mix, (no AMPerase) (Applied Biosystems, Foster City USA), 5.0 µM probe, 20 µM each primer and 2 µl of cDNA. PCR cycles were 50°C for 2 minutes, 95°C for 10 minutes followed by 60 cycles of 95°C for 15 seconds and 60°C for 1 minute. The cDNA was considered adequate quality when *GUSB* amplification was observed in all three reactions with minimal difference between crossing points.

#### TLDA

TLDA allows for the simultaneous detection of the expression of 384 genes per card. In the present study, TLDA cards were configured into four identical 96-gene sets, allowing two samples to be run in duplicate per card.

The web-based programmes *TargetScanS* (http://www.targetscan.org/) and *PicTar* (http://pictar.mdc-berlin.de/) were used to predict potential targets of *MIR-15a/16-1* for inclusion on the TLDA cards. *TargetScanS* predicts biological targets of miRNAs by searching for the presence of conserved 8mer and 7mer sites that match the seed region (nucleotides 2–7 at the 5′ end segment of the MIRNA) of each miRNA [29], while *PicTar* is a sophisticated algorithm that predicts miRNA targets by searching for pair-wise alignments that are conserved across species [30]. Genes were selected for inclusion on the TLDA cards if the following criteria were satisfied: (1) the genes were predicted as targets of *MIR-15a/16-1* by both computational programmes (*TargetScanS* and *Pictar*), and (2) Searches with Pubmed showed good biological evidence for potential involvement in tumorigenesis (Eg. proto-oncogenes, tumour-suppressor genes, transcription factors or genes involved in cell cycle regulation). Each 96-gene set included 92 predicted targets of *MIR-15a/16-1* as well as four endogenous control genes; *18S*, *ACTB*, *GUSB* and *B2M*.

For each patient sample, a reaction mix of 400 µl was prepared, including 200 µl 2x TaqMan universal master mix (no AMPerase) (Applied Biosystems, Foster City, USA), 160 µl dH_2_O and 40 µl cDNA template. Aliquots (100 µl) of the reaction mix were added to the appropriate sample loading ports on the TLDA card. The cards were centrifuged twice for 1 minute at 12,000 rpm to allow distribution of the reaction mix to each of the reaction wells. PCR amplifications were performed on the ABI 7900HT platform (Applied Biosystems, Foster City, USA). Cycling conditions were 50°C for 2 minutes, 94.5°C for 10 minutes followed by 40 cycles of 97°C for 30 seconds and 57.9°C for 1 minute. The expression of each gene was measured in duplicate for each patient.

### Analysis of gene expression

The abundance of each transcript in the TLDA analysis is directly proportional to the point in the reaction where the signal appears above the background fluorescence levels. This is termed the crossing point (Ct). Differences in the abundance of two targets are therefore directly proportional to the differences in their crossing points (ΔCt). We calculated the difference between the crossing point (ΔCt) for each test target (Ct^test^) compared with the average crossing point of four endogenous control genes (*18S*, *ACTB*, *GUSB* and *B2M*) (Ct^AveEC^) in each sample. The level of each transcript relative to that of the endogenous control levels for any given sample could then be calculated from the equation 2^−ΔΔCt^ [28] where ΔΔCt is the ΔCt value of the test transcript in that sample (ΔCt^test^) normalised to a reference transcript which was taken to be the average levels of *BCL2* in normal controls (ΔCt^ref^).

### Gene Ontology Analysis

The Gene Ontology (GO) categories of genes that were differentially expressed in CLL patients and normal controls were analysed with the ‘PANTHER Classification System’ GO browser tool. (http://www.pantherdb.org/).

### Analysis of Adenylate Uridylate-Rich Elements (ARE)

Deregulated targets of the *MIR-15a/16-1* cluster were analysed for the presence of AU-rich elements which have been reported to mediate mRNA stability (ref). Analysis was performed using the ARE-mRNA database version 3.0 (ARED; http://rc.kfshrc.edu.sa/ared/).

### Protein Expression Analysis

Where possible, protein expression analysis of the putative *MIR-15a/16-1* target genes in CLL was investigated using the ‘The Human Protein Atlas’ bioinformatics tool (http://www.proteinatlas.org/).

### Statistical Analysis

Statistical analysis was performed using SPSS 11.0 (SPSS, Inc., Chicago, IL, USA). The comparison of target gene expression between different patient groups was performed using the Mann-Whitney U test. A P value of <0.05 was considered statistically significant.

## Results

### MicroRNA-15a/16-1Expression

*MicroRNA-15a/16-1* expression was down-regulated in the majority of CLL patients (10/13, 77%), including each patient with an identified deletion at 13q14. In the majority of cases, down-regulation of *MIR-15a/16-1* correlated with chromosome 13q14 deletion status (Table 1), but in three cases (CLL 03, CLL 11 and CLL 13) the *MIR-15a/16-1* cluster was down-regulated in the absence of an identifiable 13q14 deletion.

**Table 1:** Summary of *MicroRNA-15a/16-1* expression levels and chromosome band 13q14 deletion status.

Identifier	MicroRNA-15/16-1 Expression	13q14 Deletion Status
CLL 01	Down-regulated	Deleted
CLL 02	Down-regulated	Deleted
**CLL 03**	**Down-regulated**	**Not Deleted**
CLL 04	Normal	Not Deleted
CLL 05	Normal	Not Deleted
CLL 06	Down-regulated	Deleted
CLL 07	Normal	Not Deleted
CLL 08	Down-regulated	Deleted
CLL 09	Down-regulated	Deleted
CLL 10	Down-regulated	Deleted
**CLL 11**	**Down-regulated**	**Not Deleted**
CLL 12	Down-regulated	Deleted
**CLL 13**	**Down-regulated**	**Not Deleted**

Discrepancies are highlighted in bold.

### Assessment of Sample Quality for TLDA Analysis

RNA was extracted and reverse transcribed for all subjects as described above. *GUSB* was successfully amplified in triplicate in all 18 cases (data not shown), indicating that the cDNA was of adequate quality to perform TLDA analysis.

### Micro RNA Target Prediction

A total of 99 and 145 potential gene targets were predicted by the web-based programmes (*TargetScanS* and *PicTar*) for *MIR-15a* and *MIR-16-1* respectively. Of these putative targets, 92 genes with a high likelihood for involvement in tumorigenesis were selected for inclusion on the TLDA cards.

### Differentially expressed genes in patients with CLL and Normal Controls

**Figure 1:**
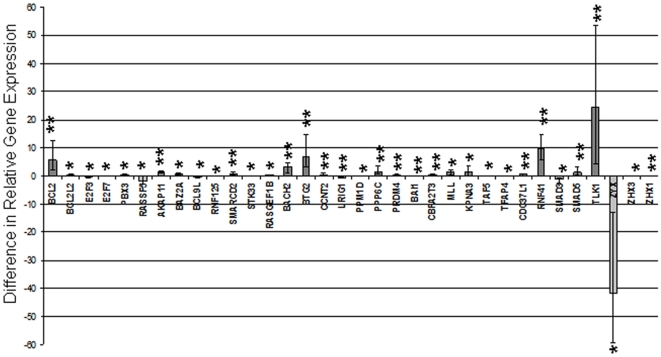
Differentially expressed genes in patients with CLL and normal controls. Bar graph representing the difference in relative gene expression in CLL patients compared with normal control patients. The dark grey bars above the x-axis represent genes that are significantly up-regulated in CLL patients compared with normal controls. Light grey bars below the x-axis represent genes that are significantly down-regulated in CLL patients when compared to normal controls. P-values; * represents <0.05, and ** represents <0.01

Real-time PCR amplification detected the expression of 80/92 (87%) target genes and all four endogenous control genes in both the CLL and the normal control patient cohorts. TLDA analysis identified 35 genes that were differentially expressed (p<0.05) between the CLL and normal control patients (Figure 1). Of these genes, 27 (77%) were up-regulated and 8 (23%) were down-regulated in the patients with CLL compared with the normal controls.

The GO of the differentially expressed genes in CLL patients and normal controls was assessed with the ‘PANTHER Classification System’ (http://www.pantherdb.org/). The most frequently represented GO categories included: (1) transcription factor, (2) cell cycle and (3) signal transduction. Other significant GO categories represented included; apoptosis, inhibition of apoptosis, nucleic acid metabolism, regulatory molecule and kinase.

### Genes specifically deregulated by the down-regulation of the MIR-15a/16-1 cluster

**Figure 2:**
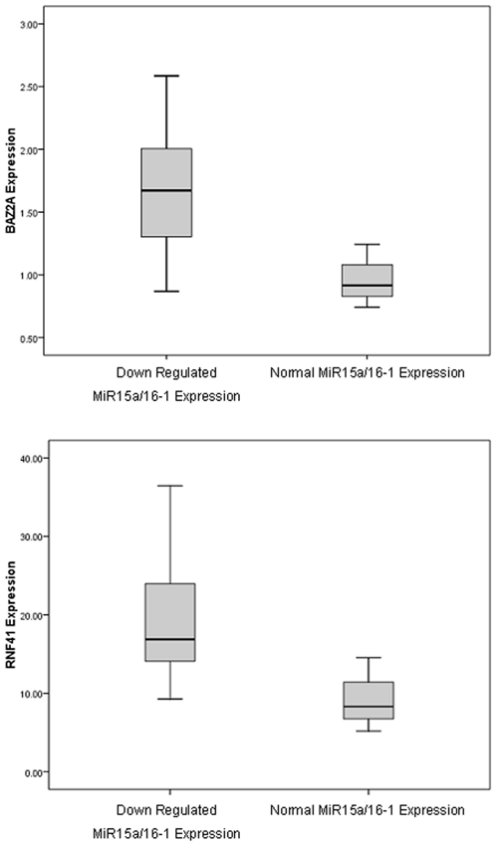
Significantly up-regulated genes in CLL patients with down-regulated *miR-15a/16-1* expression. Boxplot graphs representing the relative up-regulation of genes *BAZ2A* and *RNF41* in CLL patients with down-regulated *miR-15a/16-1* expression compared with CLL patients with normal *miR-15a/16-1* expression.

**Figure 3:**
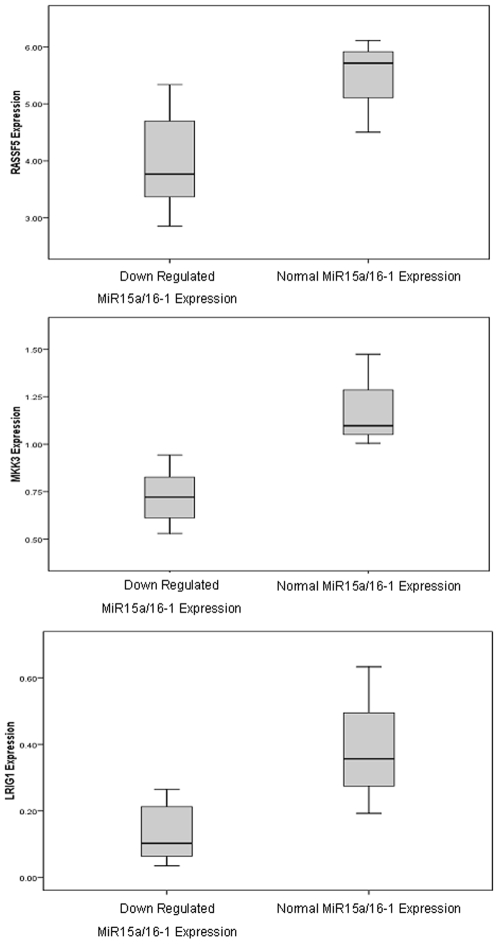
Significantly down-regulated genes in CLL patients with down-regulated *miR-15a/16-1* expression. Boxplot graphs representing the relative down-regulation of genes *RASSF5*, *MKK3* and *LRIG1* in CLL patients with down-regulated *miR-15a/16-1* expression compared with CLL patients with normal *miR-15a/16-1* expression.

Of the 92 gene targets assessed, TLDA analysis identified 5 (5%) that may be specifically deregulated by the down-regulation of the *MIR-15a/16-1* cluster. Of these 5 differentially expressed genes, 2 (*BAZ2A* and *RNF41*) were up-regulated (Figure 2) and 3 (*RASSF5, MKK3,* and *LRIG1*) were down-regulated (Figure 3) in CLL patients with low levels of *MIR-15a/16-1* expression. The putative *MIR-15a/16-1* targets were investigated for the presence of AU-rich elements, which have been implicated in regulation of mRNA stability. Of the 5 identified targets, 3 (*BAZ2A*, *RNF41* and *LRIG1*) contained AREs in their 3′ UTR.

### Protein expression analysis of the putative MIR-15a/16-1 target genes

Protein expression data from ‘The Human Protein Atlas’ (http://www.proteinatlas.org/) bioinformatics tool was available for *RNF41* and *MKK3*. This data provided evidence for the up-regulation of *RNF41* in B-CLL, since immunohistochemistry analysis demonstrated weak-moderated expression of *RNF41* in B-CLL samples, while peripheral blood mononuclear cells from healthy blood donors showed negative-weak expression of the protein. *MKK3* protein expression data showed that malignant lymphoma tissues typically exhibited weak cytoplasmic immunoreactivity or were negative. Protein expression analysis in CLL subjects for the *BAZ2A*, *RASSF5* and *LRIG1* genes is currently unavailable and represents an interesting area for future research.

## Discussion

We have investigated the expression patterns of 92 computationally-predicted targets of *MIR-15a/16-1* in 13 patients with CLL and 5 normal controls using TLDA analysis. We identified 35 genes that are differentially regulated in patients with CLL compared with normal controls and 5 genes which may be specifically regulated by the *MIR-15a/16-1* cluster at chromosome band 13q14. These genes may be important in the aetiology of CLL and as such, provide interesting targets for future studies.

A comparison of the expression profiles of CLL patients and normal controls identified 35 differentially regulated genes (Figure 1), the majority of which (77%) were up-regulated in the CLL patient group. Gene ontology analysis demonstrated that many of the differentially regulated genes were transcription factors, cell cycle-related genes or genes involved in signal transduction. Although not specifically regulated by the *MIR-15a/16-1* cluster, these deregulated genes may represent important contributors to the process of leukaemogenesis.

The 5 genes specifically regulated by *MIR-15a/16-1* expression included *2* genes (*BAZ2A* and *RNF41*) that were significantly up-regulated in CLL patients with low *MIR-15a/16-1* expression (Figure 2). These genes may represent direct biological targets of the *MIR-15a/16-1* cluster. Further experimental work including luciferase reporter gene assays or mutagenesis of predicted miRNA binding sites may be useful to examine these possibilities further. A further 3 genes (*RASSF5*, *MKK3* and *LRIG1*) were expressed at significantly lower levels in CLL patients with down-regulated *MIR-15a/16-1* expression (Figure 3). These genes may be indirect targets of *MIR-15a/16-1*, their expression perhaps being repressed by another, as yet unidentified, direct target of the *MIR-15a/16-1* cluster.

Further evidence for the deregulation of the target genes we identified in CLL was obtained from protein expression data (http://www.oriteubatkas,irg/). This analysis confirmed the up-regulation of *RNF41* in B-CLL subjects. It also demonstrated weak or negative *MKK3* protein expression in malignant lymphoma tissues, consistent with our data. The functions of the identified *MIR-15a/16-1* target genes are discussed further below. Protein data were not available for the remaining three targets we identified. This represents an interesting area for future work.

*BAZ2A* is a member of the bromodomain family of genes which function as integral components of chromatin re-modelling complexes [31]. They are believed to play a role in the chromatin-dependent regulation of transcription. Interestingly, putative *BAZ2A* deregulation has been implicated in a paediatric case of pre-B acute lymphoblastic leukemia (ALL) in which a cryptic rearrangement between 12p13 and 12q13 generated a fusion of *ETV* with an intronic sequence of *BAZ2A* [32]. The authors do acknowledge, however, that the leukaemogenic impact of putative *BAZ2A* deregulation remains undetermined at present [32].

*RNF41* is an evolutionarily conserved RING finger-containing ubiquitin ligase It has been speculated that *RNF41* is involved in the aetiology of haematological malignancies [33]. The gene resides at chromosome band 12q13, a locus that frequently demonstrates aberrations associated with acute myeloid leukemia (AML) or non-Hodgkin's lymphoma (NHL) [33]. Additionally, the gene is differentially expressed in foetal and adult haematopoietic stem cells and progenitors [33], suggesting that it may be involved in cell lineage commitment and differentiation [33]. A recent study demonstrated that over-expression of *RNF41* in a murine multipotent haematopoietic progenitor cell line (EML) attenuated erythroid and myeloid differentiation in response to the cytokines erythropoietin (EPO), interleukin-3 (IL-3) and retinoic acid [34]. This response resulted from *RNF41*-specific regulation of cytokine receptor levels [34]. Further studies are required to determine whether other haematopoietic cytokine receptors are regulated by *RNF41* and whether the gene additionally influences haematopoietic progenitor cell differentiation into lymphoid lineages.

*RASSF5* is a member of the RAS association domain family. It can act as a tumour suppressor by inducing apoptosis and delaying cell cycle progression in different cancer cell lines [35]. The gene is epigenetically silenced in a variety of human cancers by CpG island promoter hypermethylation [36], [37]. Interestingly, miRNAs can themselves act as epigenetic modifiers by the post-transcriptional regulation of chromatin modifying enzymes [38].

The mitogen-activated protein (MAP) kinase pathways mediate the transduction of extracellular signals via protein phosporylation cascades. Three distinct MAP kinase pathways have been defined; (1) extracellular-signal-related kinases (ERKs), (2) the c-Jun N-terminal kinases (JNKs) and (3) p38 stress-activated protein kinases (p38 MAPKs) [39]. *MKK3* is one of the upstream activator kinases for the p38 MAPK pathway. A recent study demonstrated that the p38 MAPK pathway, including *MKK3*, is constitutively activated in B-CLL cells but not their normal peripheral B-cell counterpart [40]. The constitutive p38 MAPK pathway activation results in up-regulation of matrix metalloproteinase-9 (MMP-9) [40], a critical factor in tumour angiogenesis and tumour homing. Elevated serum levels of MMP-9 are associated with an unfavourable prognosis for patients with CLL [41]. Our study identified significantly lower levels of *MKK3* expression in CLL patients with down-regulated *MIR-15a/16-1*. This is consistent with CLL patients harbouring chromosome 13q14 deletions, and hence *MIR-15a/16-1* down-regulation, displaying a more favourable prognosis [2].

*LRIG1* is a member of a family of *LRIG* genes that encode integral membrane proteins with extracellular/lumenal extensions consisting of leucine-rich and immuloglobulin-like domains [42]. *LRIG1* interacts with the ErbB receptor tyrosine kinase to negatively regulate EGFR signalling [43]. This regulation is mediated through the recruitment of E3 ubiquitin ligases, resulting in ubiquitinylation, internalisation and lysosomal degradation of the ErbB receptors. *LRIG1* is a proposed tumour suppressor gene. It localizes at chromosome band 3p14.3, a chromosomal region that is commonly deleted in human cancers. Additionally, *LRIG1* is down-regulated in a variety of different tumour cell lines [44] consistent with it being a tumour suppressor gene. It has been hypothesised that the down-regulation of *LRIG1* could unleash EGFR signalling which may contribute to the development of various malignancies [45]. Of note, however, *LRIG1* expression is up-regulated in some tumours, suggesting that the gene functions as a tumour promoter under certain circumstances [45]. Further studies are required to unravel the functions of the *LRIG* proteins and to further understand the contribution of *LRIG1* dysregulation to human tumorigenesis.

The majority (87/92, 95%) of the computationally-predicted targets investigated in this study were not differentially regulated in CLL patients with varying levels of *MIR-15a/16-1* expression. A possible explanation for this may be that the analysis was performed on mRNA rather than on proteins. Through imperfect pairing with their target mRNAs, some miRNAs can reduce the protein levels of a target gene with minimal variation of the mRNA levels. Alternatively, the low predictive power of the bioinformatics tools used for miRNA gene target prediction may also have contributed to this finding. Computational algorithms for the prediction of miRNA targets are acknowledged to yield a large number of false-positive hits. *TargetScanS* and *PicTar* are estimated to have a 22–31% and ∼30% false-positive rate respectively [46]. Our data suggests that these figures may under-estimate the false-positive rates associated with these programmes. Use of additional bioinformatics programmes, such as miRanda (http://cbio.mskcc.org/cgi-bin/mirnaviewer/mirnaviewer.pl), in combination may enhance the positive predictive power of these commonly used tools.

The regulation of gene expression is often complex and multifactoral. The removal of one regulatory element, such as *MIR-15a/16-1*, may be compensated for by the altered expression of other regulatory elements, thus maintaining the normal expression of the target gene. This may also explain why our study identified so few differentially regulated *MIR-15a/16-1* targets. Interestingly, the expression patterns of the anti-apoptotic gene *BCL2* may support this hypothesis. Cimmino *et al* (2005) demonstrated that *MIR-15a/16-1* negatively regulate *BCL2* [22], although this relationship remains controversial [23]. In the current study, *BCL2* was significantly over-expressed in CLL patients compared with normal controls (p = 0.001). The anti-apoptotic gene was also up-regulated in CLL patients with low *MIR-15a/16-1* expression compared to those with normal expression levels of the miRNAs, however, this did not reach the level of significance (p = 0.161) probably due to the small sample size in this study. Our data indicates that the regulation of *BCL2* may be influenced by *MIR-15a/16-1* as well as other regulatory elements, exerting a combinatorial effect.

In conclusion, our work has investigated the expression patterns of computationally-predicted targets of *MIR-15a/16-1* in patients with CLL using TLDA analysis. We have identified 35 genes that are deregulated in patients with CLL and 5 genes that are specifically deregulated by low levels of *MIR-15a/16-1* expression. The identified genes are all good biological candidates for involvement in tumorigenesis and as such, may be important in the aetiology of CLL. They provide interesting candidate genes for future studies and may represent possible targets for therapeutic intervention.
